# Diagnosis and Treatment of Hypospadias With Megameatus Intact Prepuce

**DOI:** 10.3389/fped.2020.00128

**Published:** 2020-03-31

**Authors:** Shou Xing Duan, Jianhong Li, Xuewu Jiang, Xuan Zhang, Wenhui Ou, Maxian Fu, Kaihong Chen, Lian Zheng, Shu Hua Ma

**Affiliations:** ^1^Department of Pediatric Surgery, The First Affiliated Hospital of Shantou University Medical College, Shantou, China; ^2^Department of Pediatric Surgery, The Second Affiliated Hospital of Shantou University Medical College, Shantou, China; ^3^Department of Pediatric Surgery, Shenzhen Pingshan District Woman's and Children's Hospital, Southern Medical University, Shenzhen, China; ^4^Department of Radiology, The First Affiliated Hospital of Shantou University Medical College, Shantou, China

**Keywords:** hypospadias, megameatus, intact prepuce, urethral plate, PPPS

## Abstract

**Purpose:** To evaluate the diagnosis and treatment methods of hypospadias with megameatus intact prepuce (MIP).

**Materials and Methods:** A retrospective analysis was performed in 27 MIP children, 13 of whom underwent tubularized incised plate urethroplasty (TIP procedure), 7 underwent the Duplay procedure, 5 underwent the Mathieu procedure, 1 underwent meatal advancement and glanuloplasty (MAGPI procedure), and 1 underwent the glans approximation procedure (GAP). The patients were followed for 6–36 months to evaluate the surgical outcomes by the Pediatric Penile Perception Score (PPPS).

**Results:** A total of 27 patients with a mean age of 8.12 ± 3.0 years were enrolled in this study, and 25 cases (25/27, 92.6%) were accidentally discovered during the first visit for phimosis. The patients had a formed urethra of 0.5 to 1.5 cm. Complications occurred in 4 of the 27 patients (14.81%): 2 patients with urethral fistula and 2 patients with meatal stenosis. One patient had a case of self-healed urethral fistula, and the remaining 3 patients underwent reoperation. The post-operative effect was satisfactory in all patients, and the urinary flow and stream during urination were normal. The overall average PPPS score of non-operative surgeons and parents was satisfactory. There were no significant differences in meatus appearance, glans appearance, skin appearance, and general appearance PPPS score among the Mathieu, TIP, and Duplay surgical procedures.

**Conclusions:** MIP clinical manifestations are concealed and usually noted when circumcision is attempted. The suitable procedure for each patient should be tailored according to the anatomic features, and several techniques can be used with good functional and cosmetic results.

## Introduction

Megameatus intact prepuce (MIP) is a unique variant of hypospadias and is a clinically rare condition, with an incidence of ~1/10,000, accounting for 1–3% of the incidence of hypospadias (1, 2). MIP was first reported in detail by Duckett and Keating (3), and the discovery rate of MIP is also rising with the increasing popularity of health examinations.

In recent years, an increasing number of articles have focused on this rare hypospadias variant, and the awareness of and surgical procedures for MIP continue to evolve (4, 5). Due to the anatomical particularity of MIP, it is necessary for clinicians to design a suitable surgical method taking into account the development of the glans, the width of the urethral plate, the depth of the urethral groove, and the shape and position of the urethral opening, to achieve good therapeutic effects (3, 6, 7). No single urethroplasty method provides a universal solution for all patients. In the current study, we reviewed the clinical data of 27 MIP children from our hospital to better characterize the diagnosis of and treatment methods for MIP.

## Materials and Methods

**Figure 1:**
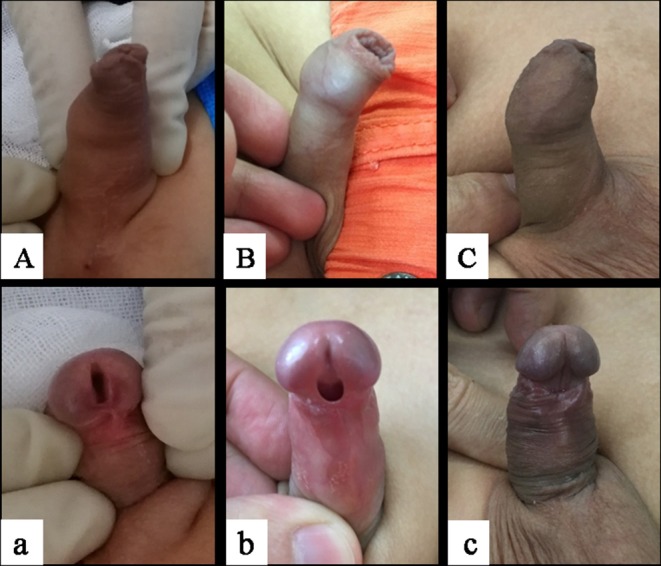
Types of megameatus intact prepuce. (**A**a) Glans, (**B**b) coronary sulcus, (**C**c) distal penile; **(A–C)** intact foreskin, (a–c) abnormal urinary meatus. The images were published with the written informed consent of the parents.

Institutional review board approval for this study was obtained from the Ethics Committee and Institutional Review Board of the Hospital of Shantou University Medical College (No. 2018-39). For the protection of privacy, the identities of the patients and physicians were scrambled in accordance with the Personal Electronic Data Protection Law. A retrospective analysis was performed for 27 children with MIP who were admitted to our hospital from January 2010 to January 2018. The ages ranged from 2 to 13 years, with an average age of 8.12 ± 3.0 years. All children with diagnosis of MIP were included. Exclusion criteria were as follows: (i) patients that have previously underwent urethroplasty/circumcision; (ii) MIP diagnosed in adulthood; (iii) Non-MIP patients with hypospadias. The first visit of the children to the hospital was as follows: 2 cases were due to abnormal position of the urethra, and the other 25 cases were discovered with phimosis during the examination before post-hetomy or exposure of the balanus during post-hetomy. Of the 27 MIP patients, the urethral opening was located in the coronary sulcus in 15 cases, the distal penis in 10 cases and the glans in 2 cases (Figure 1). Thirteen patients underwent one-stage tubularized incised plate urethroplasty (TIP procedure), 7 patients underwent one-stage penile skin tube urethroplasty (Duplay procedure), 5 patients underwent the Mathieu procedure, 1 patient underwent the glans approximation procedure (GAP), and 1 patient underwent meatal advancement and glanuloplasty (MAGPI) (Table 1).

**Table 1:** The types of MIP and the surgical techniques for the study group.

**Meatal location**	**Patients**	**TIP**	**Duplay**	**Mathieu**	**MAGPI**	**GAP**
Glans	2	0	0	0	1	1
Coronary sulcus	15	10	0	5	0	0
Distal penis	10	3	7	0	0	0

The same surgical team performed the surgeries in all cases. The urethra was sutured with 6-0 absorbable thread, and proper pressure was applied to the penis with a self-adhesive elastic bandage. Antibiotics were used to prevent infection after surgery. Urethral catheters were in place for 12–14 days, and the patients were followed for 6–36 months.

The surgical results were evaluated according to the non-operative surgeons and parents' satisfaction with the appearance of the penis after surgery and the urination of the child. Satisfaction with the appearance of the penis after surgery was assessed with reference to the Pediatric Penile Perception Score (PPPS) (8, 9). The specific scoring items include the appearance of the meatus, the appearance of the glans, the appearance of the skin, and the general appearance. Each item is scored according to the subjective satisfaction of the subject: very satisfied (3 points), satisfied (2 points), dissatisfied (1 point), and very dissatisfied (0 points) (8, 9). The outcomes were compared in the different groups. One-way analyses of variance (ANOVA) were adopted for statistical analysis, assuming *p* < 0.05 as significant.

## Results

**Figure 2:**
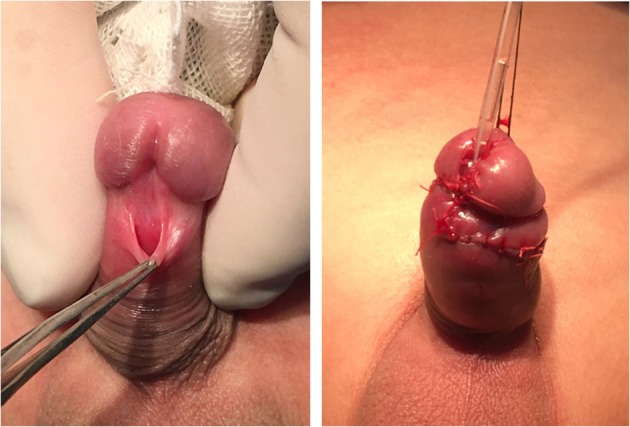
The giant urethral opening was reduced and translocated to the middle of the glans. The images were published with the written informed consent of the parents. The patient is the same case as Figure 1C,c (Supplementary Figure 1).

The urethras formed in the 27 MIP patients ranged from 0.5 to 1.5 cm (Figure 2). One patient underwent MAGPI, one underwent the GAP, 5 underwent the Mathieu procedure, 13 underwent the TIP procedure, and 7 underwent the Duplay procedure. Complications occurred in 4 of the 27 patients (14.81%): 2 patients with urethral fistula and 2 patients with meatal stenosis. Urinary fistula occurred as early as 2 weeks after surgery, and meatal stenosis occurred 48 weeks after surgery. There was 1 case of self-healed urethral fistula, and the remaining 3 patients underwent reoperation (Table 2).

**Table 2:** The complications of the surgical procedures.

**Surgical procedures**	**Patients**	**Complications**	**Cure rate**	**Fistula**	**Meatal stenosis**	**Diverticulum**	**Infection**	**Reoperation**
TIP	13	2	11/13	1	1	0	0	1
Duplay	7	1	6/7	0	1	0	0	1
Mathieu	5	1	4/5	1	0	0	0	1
MAGPI	1	0	1/1	0	0	0	0	0
GAP	1	0	1/1	0	0	0	0	0

**Figure 3:**
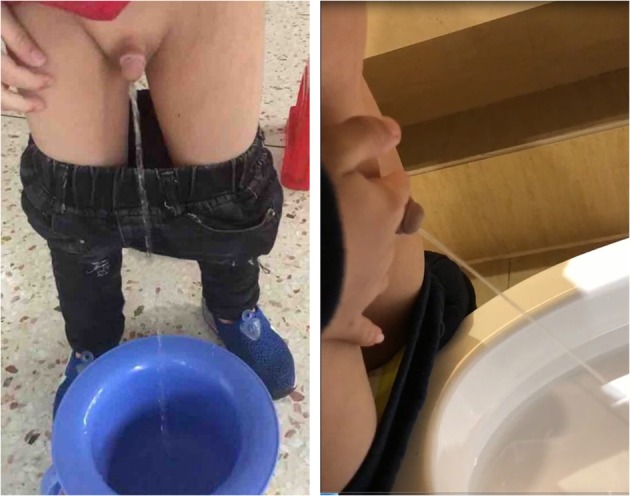
Postoperative follow-up showed satisfactory appearance and normal urinary flow and stream. The images were published with the written informed consent of the parents.

The post-operative effect was satisfactory in all patients, and the urinary flow and stream during urination were normal. There was no urinary fine line or dysuria, no urine flow spray, and no urinary fistula or other complications (Figure 3). According to the PPPS score, non-operative surgeons and parents had a satisfaction score for the meatus appearance, glans appearance, skin appearance, and general appearance. One-way ANOVA was used to compare the statistical differences of these PPPS score among the Mathieu, TIP, and Duplay surgical procedures, and the results showed no significant differences (*P* > 0.05) (Table 3).

**Table 3:** PPPS scores of urinary surgeon and parents of children with MIP among different surgical procedures^*^.

**Surgical procedures**	**Meatus appearance**		**Glans appearance**		**Skin appearance**		**General appearance**	
	**Parent's score**	**Urinary surgeon's score**	**Parent's score**	**Urinary surgeon's score**	**Parent's score**	**Urinary surgeon's score**	**Parent's score**	**Urinary surgeon's score**
Mathieu	2.60 ± 0.89	2.80 ± 0.45	2.40 ± 0.89	2.80 ± 0.45	2.60 ± 0.55	2.80 ± 0.45	2.40 ± 0.89	2.80 ± 0.45
TIP	2.23 ± 0.44	2.31 ± 0.48	2.23 ± 0.73	2.62 ± 0.51	2.15 ± 0.55	2.54 ± 0.52	2.31 ± 0.63	2.46 ± 0.52
Duplay	2.29 ± 0.63	2.43 ± 0.53	2.43 ± 0.79	2.71 ± 0.49	2.14 ± 0.69	2.43 ± 0.53	2.43 ± 0.98	2.43 ± 0.53
F	0.62	1.83	1.80	0.28	1.15	0.79	0.61	0.95
P	0.55	0.19	0.84	0.76	0.34	0.46	0.94	0.40

* *Excludes the procedures with only one patients data (MAGPI and GAP procedures)*.

*Scores correspond to satisfaction: very satisfied (3 points), satisfied (2 points), dissatisfied (1 point), and very dissatisfied (0 points)*.

## Discussion

The embryologic pathogenesis of MIP still remains unclear. Duckett and Keating (3) has suggested that the foreskin and urethra develop independently and are unrelated. Due to excessive division of the glans, the distal urethra that has already formed is split to form a large urethral opening, while the foreskin develops normally. Nonomura et al. (10) speculated that ischemia and compression necrosis may occur after formation of a normal urethra, causing MIP. Stephens and Fortune (11) considered that the ingrowth of the epithelium on the top of the glans leads to delayed connection or failed fusion with the proximal urethra, which results in temporal high-pressure blockage of the distal urethra, thus forming MIP. It has been theorized that hypospadias results from incomplete fusion of the urethral folds, resulting in an incomplete urethra and incomplete or hooded foreskin. In the MIP variant of hypospadias, glanular urethra forms from ectodermal pit at glans tip and open end of urethral groove. Maldevelopment of glanular epithelial infolding would appear to be the abnormal process responsible for MIP formation. Complete closure of urethral fold and prepuce, but canalization of glanular plate is incomplete that leads to megameatus intact prepuce (5, 12, 13). Until now, all of these theories have failed to explain the embryology of MIP, and the specific reasons need further exploration.

The MIP lesion is hidden and difficult to find; most patients are often misdiagnosed early as having phimosis because the foreskin is intact. Some cases of MIP are found during circumcision in neonates or infancy in Europe or America (14). In the current study, 25 cases were accidentally discovered during a visit for phimosis, while the family members were unaware of any urinary tract abnormalities. Abnormalities were found during circumcision, which was then changed to surgery for hypospadias. The actual incidence of MIP might be much higher than what is reported because a significant number of children with MIP are not detected or are untreated after diagnosis.

The distinct anatomic features of MIP have led it to differ from other typical hypospadias. The anatomic characteristics of MIP are as follows: an intact foreskin, wide and fish-like urethral opening, wide and shovel-like glans, deep navicular fossa, and no ventral downward curvature or just slight dorsal bending of the penile body (7). MIP can be divided into glans, coronary sulcus and distal penis types according to the position of the urethral opening (7). For the particularity of MIP anatomical morphology, it is necessary for clinicians to design a suitable surgical method taking into account the development of the glans, the width of the urethral plate, the depth of the urethral groove, and the shape and position of the urethral opening to achieve good therapeutic effects (6, 15).

It still remains controversial whether patients with partial MIP, but with normal micturition and an unobstructed sexual life and those whose daily life is unaffected must undergo surgical intervention. These patients can choose instead a conservative treatment (12). However, surgical correction of MIP in the era of increased cosmetic awareness is justified. The purpose behind MIP treatment is to reduce the giant urethral opening and move the urethral opening to the middle of the glans.

For the glans type of MIP, MAGPI or the GAP procedure can achieve good surgical results in order to restore the morphology and function (6, 16). These procedures can overcome the challenges of a wide, deep glanular groove and a non-compliant fish mouth. In our series, two patients with glanular MIP underwent MAGPI and the GAP procedure. The post-operative results were satisfactory, and there were no complications. For the coronary sulcus or distal penis type of MIP, the urethral plate should be retained during correction because of the lack of an obvious penile curvature, and the Mathieu, Duplay or TIP procedures can be performed. The Mathieu technique uses a reverse proximal urethral flap to match the urethral orifice, retaining the distal urethral plate, with no distal or proximal urethral anastomoses, thus reducing the occurrence of urethral stricture (12). The patients who underwent the Mathieu procedure no meatal stenosis occurred except for one case of urinary fistula.

The Duplay procedure is suited for patients with a wider urethral plate that can be directly rolled up to complete the urethral formation (7). Because the urethral plate retains a good blood supply and there is no annular anastomosis, the occurrence of a urethral stricture or urinary fistula is significantly reduced. The patients in our study who underwent the Duplay procedure had a fascia flap, with a rich blood supply, applied to cover the formed urethra, further reducing the incidence of post-operative urinary fistula. One of the patients who underwent the Duplay procedure developed a meatal stenosis, which may be associated with urethral scar hyperplasia and contracture.

If the patient has a general wide or a usual urethral plate, the TIP procedure seems to be a good alternative treatment. The TIP procedure can result in a good penile appearance and functional effects for the treatment of distal hypospadias, as well as a very low complication rate (17, 18). In our study, 13 patients underwent the TIP procedure, one of whom needed reoperation for repair. The success rate of the one-time TIP procedure was 92.6% (12/13) (19). To better reduce the occurrence of a urinary fistula in the TIP procedure, we found that the U-shaped incision of the urethral plate around the urethral opening should be over the urethral cavernosum surface to avoid damage to the cavernosum and bleeding. The membranous urethra and the urethral plate peripheral skin had to be fully resected to facilitate healing of the sewn urethra. The midline incision of the urethral plate should be over the corpus cavernosum surface to prevent bleeding, and the urethral plate should be fully expanded to ensure that the urethral suture is tension-free (20). In addition, we used the subcutaneous fascia to cover the urethra, which significantly reduces the incidence of urinary fistula (21, 22).

Our satisfaction score results show that there were no significant differences in meatus appearance, glans appearance, skin appearance, and general appearance PPPS score among the Mathieu, TIP, and Duplay surgical procedures. These procedures have good therapeutic effects on MIP. The Mathieu procedure is suitable for cases where the urethral plate is not wide enough and the formed urethra is short. The Duplay procedure can be used in patients with wider urethral plates that can be directly rolled up for complete urethroplasty. The TIP procedure can be used for a general wide or a usual urethral plate MIP and is also suitable for cases where initial surgery fails and there is a need for reoperation (23).

According to the PPPS score (9), the overall average PPPS score of non-operative surgeons and parents was satisfactory. The post-operative effect was satisfactory in all patients, and the urinary flow and stream during urination were normal. The high success rate of these operations may be due to the normal blood supply of the urethral plate and the urethral covering of the fascia; the flat urethral plate supported by the sponge can provide a fixed platform for the newly formed urethra. Histological studies of the urethral plate have found that the urethral plate, with a rich blood flow supported by the corpus cavernosum, contains abundant vascular smooth muscle, glands and nerves, and its smooth muscle and connective tissue have strong extensibility. These characteristics are very beneficial for urethral reconstruction (23, 24). The MIP anatomical features of a wide urethral plate, deep navicular fossa, and large glans create favorable conditions for these three surgical methods.

## Conclusion

**Figure 4:**
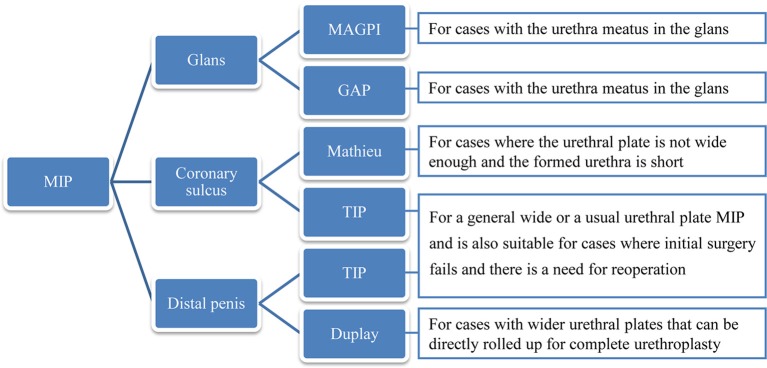
The detailed flowchart of the decision can be driven according to the anatomy of the specific case.

In conclusion, MIP clinical manifestations are concealed and usually noted when circumcision is attempted. The suitable procedure for each patient should be tailored according to the anatomic features, and several techniques can achieve good cosmetic and functional results. For the glans type of MIP, both MAGPI and GAP procedures can achieve good surgical results; for the coronary sulcus or distal penis type of MIP, the Mathieu, Duplay, or TIP procedures can be performed. The choice of a particular technique can follow the process as presented in the flowchart (Figure 4), taking into consideration the anatomy of the specific case to drive the decision-making process.

## Data Availability Statement

The datasets for this article are not publicly available because the patients' files are publicly available but information was collected for scientific purposes. Requests to access the datasets should be directed to Shouxing Duan, duanshouxing@126.com.

## Ethics Statement

Ethical approval for this retrospective study was obtained from the Ethics Committee and Institutional Review Board of the Hospital of Shantou University Medical College (No. 2018-39).

## Author Contributions

SD: data collection, wrote, and corrected the manuscript. SD and LZ: data analysis and manuscript preparation. JL, XJ, XZ, WO, MF, and KC: performed surgery and data collection. LZ and SM: study idea and revised the manuscript. All authors listed have made a substantial, direct and intellectual contribution to the work, and approved it for publication.

### Conflict of Interest

The authors declare that the research was conducted in the absence of any commercial or financial relationships that could be construed as a potential conflict of interest.

## Supplementary Material

Supplementary Figure 1 The patient of Figure 2 is the same case as Figure 1C,c.
