# Supplementary figures and images for: Diagnosis and Treatment of Hypospadias With Megameatus Intact Prepuce
Source: Front Pediatr. 2020 Mar 31;8:128. doi: 10.3389/fped.2020.00128 (PMC7136419; doi:10.3389/fped.2020.00128)

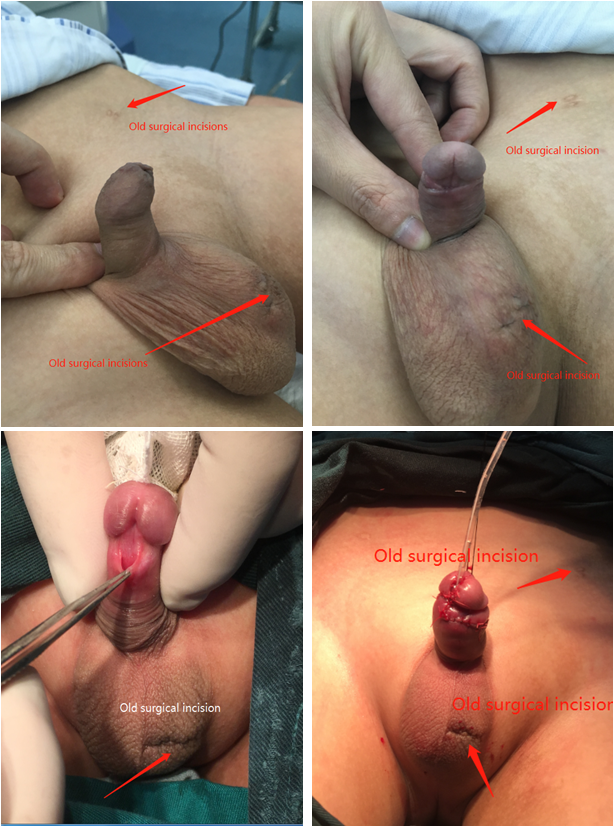

Supplement: Supplementary Figure 1 — The patient of Figure 2 is the same case as Figure 1C,c. [file Image_1.TIF]
